# Inequalities, eating practices and beliefs among transgender women in Colombia: Mixed approaches in research

**DOI:** 10.1016/j.dialog.2026.100287

**Published:** 2026-02-26

**Authors:** Ana Lucia Valenzuela-Gallego, Paula Andrea Hoyos-Hernández, Laura Lucia Dominguez-Barrios, José Rafael Tovar-Cuevas

**Affiliations:** a Facultad de Ciencias de la Salud, Pontificia Universidad Javeriana, Cali, Colombia; b Facultad de Humanidades y Ciencias Sociales, Pontificia Universidad Javeriana, Cali, Colombia; c Universidad del Valle Cali, Cali, Colombia

**Keywords:** Eating practices, Trans women, Health inequalities, Structural barriers, Qualitative analysis, Bayesian paradigm

## Abstract

This study aimed to describe and categorize the eating practices and beliefs of trans women using a mixed-methods approach. Conducted in Cali, Colombia, the study included 33 trans women aged 18 years or older who voluntarily participated in August 2021. Food consumption and eating practices were assessed through structured questionnaires, and the data were analyzed using a Bayesian statistical approach and the Healthy Eating Practices Index (HEPI). In addition, discussion groups were conducted to explore participants' experiences and food-related beliefs. The results showed that 55% of the trans women presented low levels of healthy eating practices, characterized by limited dietary diversity in recommended food groups and a higher frequency of consumption of less recommended foods. Being under 30 years of age, belonging to a middle socioeconomic level, having lower levels of education and income, living with a partner, and regularly having a salt shaker on the table were factors individually associated with a higher likelihood of unhealthy eating practices. The multiple inequalities faced by the transgender population generate structural barriers that shape food practices within the community as adaptive responses to their living conditions, perpetuating unfavorable dietary patterns. These findings highlight the need for primary health care strategies with a gender-sensitive and trans-affirmative approach, in which nutrition and food practices are addressed through the lens of the social determinants of health. Transforming hegemonic social imaginaries is essential for building inclusive health environments that respect and reflect human diversity.

## 1 Introduction

Historically, hegemonic models have generated unequal and exclusionary social relations, which have been harsher for the trans population, who have experienced multiple violations of their human rights due to transphobia, sexual violence, murder, and social, labor, and family exclusion [1]. In Colombia, this reality persists, and trans women, in particular, suffer the most severe cases of discrimination, stigma, poverty, precarious employment, psychological, physical, and sexual violence, and murders related to their gender identity [2,3].

In addition, Colombia has gaps in healthcare access for this population, linked to a limited institutional capacity for transgender care, coupled with barriers to access to safe hormone therapies from an early stage and healthcare treatments tailored to their needs. Furthermore, mistreatment, various forms of violence, and discrimination by healthcare personnel persist in these contexts [1,[4], [5], [6]].

Within the context of the health of the trans community, a topic that has been little explored is food and its nutritional aspects [[7], [8], [9]]. Some of the studies that have been carried out claim that food and nutrition in trans people offer a perspective related to food care derived from hormone therapy. Another examines food with psychosocial aspects that may impact the trans community's mental health, food consumption, and the possible generation of eating disorders that affect their nutrition [[9], [10], [11]].

On the other hand, although studies are scarce, one ethnographically designed study found that digital platforms are used as an informal means of exchanging experiences and nutritional knowledge among trans people. For example, the messages that have been shared addressed the use of supplements, weight management, body shaping, and nutrition during hormone therapy. These findings are relevant to understanding emerging dietary practices during gender transition, offering an opportunity to design nutritional strategies that incorporate both traditional knowledge and scientific evidence [11]. The support of health professionals on these websites is considered essential to building processes that integrate diverse knowledge, including scientific evidence. Paradoxically, there is a severe lack of theory and methodology among nutrition professionals to provide scientific advice to the trans population [8,12].

In Colombia, no studies have been carried out on the eating practices of the trans population. However, the literature states that, despite this gap, recommendations should address the community's needs regarding hormone therapy, the risks of disease, and other factors identified as meriting nutritional intervention [13].

In this study, eating practices were considered behaviors for obtaining, selecting, preparing, and consuming food across various interactions within a particular context. These are biologically important because they provide the body with energy and nutrients. However, they are formed and configured in complex processes, not only by the food culture of an individual's upbringing, but also by other sociocultural, economic, psychological, religious, and educational factors that can change during a person's life [14,15].

Inherited beliefs and practices also create habits, as beliefs are woven into precepts, taboos, and/or meanings that confer value on food, thereby cataloging what may be adequate or inadequate for health or other conditions [16]. However, scientific evidence has standardized the most and least healthy eating practices, which are associated with different variables related to disease prevention. Colombia has the Food-Based Dietary Guidelines for the Colombian Population (Spanish acronym GABA). This national reference aims to promote a complete, balanced, sufficient, and adequate diet to reduce malnutrition due to either deficiency or excess, promote health, and decrease the development of non-transmissible diseases.

To achieve this goal, it is recommended to frequently consume a diet that includes natural and diverse food items from various food groups, such as whole grains, tubers, roots and plantains, fresh fruits and vegetables, legumes, dairy products, a variety of protein-rich foods, and foods rich in essential and monounsaturated fatty acids. Conversely, it is recommended to reduce excess salt, ultra-processed foods, fast food, saturated fats, and sugary drinks. These recommendations constitute a fundamental principle of healthy eating practices and apply to the general population. However, they do not include specific recommendations for the transgender population; therefore, the recommendations are adapted according to the needs identified within this population [[14], [15], [16], [17]].

Currently, no studies have been identified in Colombia that specifically analyze the structural barriers faced by transgender women related to food or the adoption of a healthy diet. However, national and international literature has extensively documented the multiple vulnerabilities they face; therefore, these conditions allow us to hypothesize a negative impact on food security and nutritional state.

The described context highlights the lack of research that integrates the food and nutritional component of trans women, given the limited knowledge about the dietary practices and beliefs they develop within the framework of their gender transitions. Therefore, this research aimed to describe and categorize the practices and beliefs of a group of trans women residing in Cali, Colombia. Based on this exploratory approach, the study seeks to contribute to understanding this population by generating conceptual and empirical insights that can guide the development of future nutritional interventions with a differential approach.

## 2 Method

### 2.1 Design

A mixed-methods approach with a convergent parallel design was employed, using a qualitative, phenomenological, and interpretative methodology (triangulation scope 12) and a cross-sectional, observational, and quantitative analytical method [18,19].

Although the study intentionally incorporated both components, quantitative analysis carried greater analytical weight, given the robustness of the data collected and the development of descriptive analyses, the construction of the Healthy Eating Practices Index (HEPI), and the Bayesian estimation of predictive probabilities. The qualitative component was not directly integrated into the statistical analysis, but rather served a complementary function by providing contextual and interpretive elements about the participants' dietary beliefs. The results were mainly integrated into the discussion, where the qualitative findings allowed for a broader understanding of the quantitative patterns, without attempting to establish a direct or explanatory correspondence between the two sets of data. The final interpretation of the study was therefore constructed from a logic of complementarity between approaches, consistent with an exploratory mixed-methods design.

### 2.2 Participants

Thirty-three trans women aged 18 or older participated through purposive sampling. The inclusion criteria were a) being over 18 years old, b) self-determining as a trans woman, and c) residing in the city of Cali. No exclusion criteria were considered. The sample was established using the chain sample technique or networks, with the support of trans women leaders in the city.

### 2.3 Procedure

This study was carried out in 4 phases from May to August 2021. In the first contact phase, a network was formed with trans women leaders from Cali participating in the Macro Project (this Project was a participatory action research that was carried out between February 2019 and February 2022), and a social worker with experience in the trans population, who in turn communicated to their networks of trans women about the project. The second phase involved reaching out to potential participants and was carried out by telephone between the researchers and the trans women interested in participating. The third phase involved data collection and involved 33 participants. Four groups met on different days, in a specific place with the necessary materials and instruments. Informed consent was obtained from all participants. Subsequently, the “circuit” strategy was used to collect data. The fourth and final phase consisted of data analysis and the delivery of results using the software R version 4.1.2 for Windows and IBM SPSS Statistics. Once the individual and group reports were consolidated, the results were given to the participating women, governmental and non-governmental institutions, professionals, and social science and health students.

### 2.4 Instruments

For the quantitative component, a structured interview on sociodemographic, health, and dietary aspects was used. An ad hoc adapted version of the research by Hoyos-Hernández et al. [8] was used the Encuesta Nacional de Situación Nutricional (ENSIN) [20]. Structured Food Consumption Frequency Questionnaire (SFCFQ) was also used, which has been piloted and applied in two national surveys (2010: 50,670 households; 2015: 44,000 households). It consists of 48 items, of which 36 correspond to food consumption frequencies and food groups (4 items measuring general consumption practices and 8 items measuring consumption times). The scale measures the proportion of monthly consumption using a dichotomous variable (yes/no). Then it classifies habitual intake frequency into 10 categories, ranging from daily to monthly or less than once a month. Thus, it enables approximating healthy and unhealthy consumption patterns and eating practices.

The SFCFQ/ENSIN public use instrument was applied in its original version and did not require a license for academic use. Although no specific pilot test was conducted in the transgender population, the instrument has been piloted and validated at a national level through population surveys in Colombia (ENSIN 2010 and 2015). Prior to data collection, a training and standardization process was carried out with the research team to ensure consistent application.

Regarding the qualitative component, four discussion groups were conducted with the same women who participated in the quantitative component. The groups were guided by a semi-structured interview protocol covering: a) socio-demographic characteristics, b) culinary and dietary practices, and c) food beliefs and practices related to gender transition. These pre-established categories were defined by the research team, which incorporated a leading trans researcher. The saturation of information across the analysis categories was achieved with 22 participants [21]. The data facilitated a phenomenological analysis of eating practices and beliefs among transgender women in Cali, Colombia.

### 2.5 Data analysis

When conducting the statistical analysis, two methodological aspects were considered: the characteristics of the sample and the multidimensional configuration that constitutes the degree of the individual's healthy eating practices. Understanding this degree as a latent variable, the individual's responses regarding the food she eats were treated as observable variables. Thus, 29 items representative of the SFCFQ and their consumption frequency (daily, weekly, monthly) were used, of which 20 were classified as healthy and 9 as unhealthy, according to the recommendations of the FBDG [14].

To obtain a numerical approximation of the extent to which healthy eating practices are present or absent, an index (Healthy Eating Practices Index – HEPI) was constructed rather than applying traditional diet quality indices. This decision was based on the need to integrate dietary variables supported by the Food-Based Dietary Guidelines (Spanish acronym GABA) for Colombia, along with sociodemographic variables relevant to the trans population. The HEPI was developed for exploratory purposes, given the limited available literature and the interest in understanding, in a contextualized manner, the eating practices of a historically difficult-to-access population, with a small sample size. The proposed HEPI allows for the description of eating practices, so that, through a numerical value on a scale of 0 to 100, the degree to which these eating habits are present in individuals, in this case, members of the trans community, can be quantified. Based on the information collected, the HEPI has a theoretical range of −1.7 to 2.7, which was transformed into a percentage scale and classified using a cutoff point of 70% for descriptive and analytical purposes. The index value was obtained for each participant, and, using a heuristically developed classification rule and expert input, it was determined whether the individual exhibited a high or low level of presence [22].

### 2.6 Constructing the Healthy Eating Practices Index (HEPI)

The HEPI is based on the theory of latent variables, as defined by the Food-Based Dietary Guidelines for Colombia (GABA) and published by the Colombian Institute of Family Welfare (ICBF) [14], as well as the referents of Losada and Bidau [15]. The level of healthy eating practices is assumed to be a characteristic that is not directly observable. Therefore, it should be treated as a synthetic variable, quantified through an index, once the indicator variables have been operationalized [24]. The HEPI was designed using the items that allowed the variable to approach a measurable characteristic. 29 food groups were selected, 20 of which are healthy, and the remainder were classified as unhealthy (Table 1).

**Table 1:** Healthy and unhealthy food groups were used in the design of the HEPI.

Food groups		Coding^a^
*Healthy eating practices*		
Dairy and dairy products	Milk (liquid or powdered) by itself or in mixtures	$\mu_{1}$
	Cheese, kumis, yogurt and related products	$\mu_{2}$
Sources of animal protein	Eggs	$\mu_{3}$
	Chicken	$\mu_{4}$
	Beef, veal, pork, capybara	$\mu_{5}$
	Fish or Seafood	$\mu_{6}$
	Tuna or sardines	$\mu_{7}$
	Chicken giblets	$\mu_{8}$
	Black pudding o beef entrails (liver, liver, lung, kidney, etc.)	$\mu_{9}$
Sources of vegetable protein	Dried pulses (beans, peas, chickpeas, lentils, lima beans, soybeans)	$\mu_{10}$
	Rice and pasta	$\mu_{11}$
Cereales and related products	Bread	$\mu_{12}$
	*Arepa* (Derived from corn)	$\mu_{13}$
	Cookies	$\mu_{14}$
	Whole grain foods (bread, rice, cookies, etc)	$\mu_{15}$
Tubers and plantains	Tubers and plantains (potato, cassava, yam, plantain)	$\mu_{16}$
Vegetables	Cooked vegetables	$\mu_{17}$
	Raw vegetables	$\mu_{18}$
Fruit	Fruit juices^b^ Whole fruit	$\mu_{19}$ $\mu_{20}$
Unhealthy eating practices	Sausages (sausage, salami, ham, sausage)	$\pi_{1}$
	Butter, cream, lard, cream cheese, coastal whey	$\pi_{2}$
	*Panela* (derived from sugar cane), sugar or honey	$\pi_{3}$
	Candies or sweets	$\pi_{4}$
	Soda, tea and soft drinks (powdered, boxed, bottled); Non-light	$\pi_{5}$
	Consumption of alcoholic beverages (any type of product, straight or mixed)	$\pi_{6}$
	Fast food (hamburgers, hot dog, pizza, tacos, etc.)	$\pi_{7}$
	Packaged foods (potato chips, etc.)	$\pi_{8}$
	Fried food (french fries, fried meat, fried plantains, etc.)	$\pi_{9}$

μ identifies healthy food groups, and π represents unhealthy food groups. Both take the value of one (π = μ = 1) when the participant claims to consume the food group and (π = μ = 0) when she does not.

a Mathematical symbolic identification used in the equations within the mathematical procedure of index design.

b The FBDG 2020 recommends consuming a diverse diet that includes several food groups (according to the characteristics of the Colombian population), which includes fruit. The FBDG document does not prohibit the consumption of juiced fruits; it recommends whole fruits for greater health benefits. The fruit juices may contain added sugars and contribute to a low fiber intake.

Information about the frequency of food consumption was also screened. The instrument used three response categories (Daily, Weekly, and Monthly), each assigned a value of 1, 2, or 3 depending on the type of food. Healthy foods or a healthy eating habit would be associated with daily frequency, in which case this category would take the value 3. For unhealthy foods, under the same hypothesis (healthy habit), it was expected that their consumption frequency would be low (monthly, with a value of 1).

For the construction of the index indicators, information on the following variables was included:

$X_{1}$: Total number of meals that the person ingests among the 29 proposed.

$X_{2}$: Number of healthy meals that the person ingests among the 20 proposed.

$X_{3}$: Number of healthy meals that the person ingests among the nine proposed.

The following indicators were constructed to serve as observed variables:

$\lambda_{1}=\frac{X_{1}}{29}$ Proportion of food groups reported by the individual among those proposed.

$\lambda_{2}=\frac{X_{2}}{20}$ Proportion of health-promoting food groups that the individual reports consuming among those proposed.

$\lambda_{3}=\frac{X_{3}}{9}$ Proportion of unhealthy food groups that the individual reports consuming among the proposed ones.

$\alpha_{1}=\frac{X_{2}}{X_{1}}$ Proportion of health-promoting food groups among the food groups consumed by the subject.

$\alpha_{2}=\frac{X_{3}}{X_{1}}$ Proportion of unhealthy food groups among the food groups that the subject consumes.

Consuming foods from the food group was valued as one and for each participant, the product between the presence of consumption and its frequency (β) was made. With the products, two new variables $Z_{1}$ and $Z_{2}$:

(1) $$Z_{1}=\sum_{j=1}^{X_{2}}\mu_{j}\beta_{j};Z_{2}=\sum_{k=1}^{X_{3}}\pi_{j}\beta_{k}\;$$

Where: $\beta =1,2,3;j=1,\ldots .,X_{2};k=1,\ldots \ldots X_{3};\pi ,\mu =0,1$

When carrying out addition for products, it is expected that, $X_{2}\leq Z_{1}\leq 3X_{2}$ and $X_{3}\leq Z_{2}\leq 3X_{3}$. An individual has an adequate diet if their responses add up to just $Z_{1}$ ($Z_{1}>0$ and $Z_{2}=0$), which would be the ideal situation. At the other extreme, is the person who only consumes food that is not very good for their health ($Z_{1}=0$ and $Z_{2}>0).$ The two situations are the extremes on a continuum in which different nuances can be identified, since an individual can include both healthy and unhealthy foods in their diet with any of the three frequencies for each case.

Since the numerical scales of the variables $Z_{1}$ and $Z_{2}\;$take different value ranges in real numbers, it was standardized so that both would be expressed in the interval $\left(01\right)$, using equation two:

(2) $$Z_{1}^{*}=\frac{Z_{1}-X_{2}}{3X_{2}-X_{2}}=\frac{Z_{1}-X_{2}}{2X_{2}};Z_{2}^{*}=\frac{Z_{2}-X_{3}}{3X_{3}-X_{3}}=\frac{Z_{2}-X_{3}}{2X_{3}}.$$

Based on the values taken by the variables $Z_{1}^{*}$ and $Z_{2}^{*}$, it was heuristically established that (based on the healthy foods consumed) an individual has an adequate (healthy) diet when $Z_{1}^{*}\geq 0.51$. Finally, the equation for the ${\mathrm{IHEP}}_{i}^{*}$ of an individual *i* is eq. (3) and in theoretical terms it is expected be, −1.7≤IHEP≤2.7

(3) $$\mathrm{IHEP}=\lambda_{1}+\lambda_{2}-\lambda_{3}+\alpha_{1}Z_{1}^{*}-\alpha_{2}Z_{2}^{*}.$$

For operational and practical purposes, a change of scale was made using eq. (4), so that $\mathrm{IHEP}\;\epsilon \;\left(0,100\right).$Based on the hypothesis that an adequate diet is present when more healthy than unhealthy foods are consumed with very often (which implies that the relationship between the variables constructed is $\alpha_{1}Z_{1}^{*}>\alpha_{2}Z_{2}^{*}$), a cut-off point was defined for the HEPI in order to classify the presence of adequate eating practices on two levels. It was defined that, if the HEPI takes a value greater than or equal to 70, the individual has very healthy eating practices.

(4) $${\mathrm{HEPI}}_{I}^{*}=\frac{\mathrm{HEPI}+1,7}{4,4}*100\;$$

Given the limitations of the sample in terms of size and the lack of a randomization mechanism for participant selection, it was decided to conduct the data analysis using Bayesian statistical procedures. Making inferences this way does not require the sample to consist of independent and identically distributed observations, as a classic analysis does. The assumption of independence can also be relaxed by assuming permutability in the sample composition. When faced with a small sample size, the use of information external to the sample (placed in the form of a probability distribution), strengthens the results obtained in the inferential process. Predictive Bayes probabilities were calculated to estimate the probability that a trans woman would have high levels of eating practices relating to sociodemographic characteristics and practices associated with eating that may affect health. Such probabilities can be used as risk indicators by decision-makers and public health professionals through data analyses in R.

It should be noted that the information was collected in person by the research team, which minimized recording errors and ensured the consistency of the database. For the statistical analyses, a descriptive analysis was performed of the frequencies of consumption of healthy and unhealthy foods, as well as the sociodemographic characteristics of the participants. To evaluate the association between the adequate eating habits index and sociodemographic variables, the Chi-square or Fisher's exact tests were used, depending on the fulfillment of statistical assumptions, considering a significance level of 5%. No imputation of missing data was performed; the analyses were carried out with the complete information available for each variable. Possible selection and clustering biases derived from chain sampling are acknowledged and explicitly addressed in the limitations section. The study is reported in accordance with the recommendations of the STROBE guidelines for observational studies, whose checklist is included as supplementary material.

For the Bayesian estimation of predictive probabilities, a non-informative Beta prior distribution (α = 1, β = 1) was assumed, selected to allow the observed information to dominate the posterior inference. The approach used corresponds to a binomial-beta model with a closed analytical solution, so there was no need to implement stochastic simulation algorithms or evaluate convergence diagnostics. No formal sensitivity analyses were performed with respect to the specification of the prior distribution; consequently, the results derived from the Bayesian approach should be interpreted within an exploratory and contextual framework, consistent with the sample size and scope of the study.

The analysis of the qualitative data was performed through thematic analysis [23] using Atlas. ti 22, which considered the saturation of the categories [21]. Consensual Qualitative Research (CQR) was conducted to ensure a reliable transfer of the women's experiences to the contexts in which they occurred. The categories were constructed from conversations with the women. To ensure rigor, the coding was carried out separately by two researchers and later validated by a community leader. The findings are presented in the results section.

Interviews and discussion groups were conducted by researcher and a community co-facilitator, who is a psychologist with expertise in qualitative methods, gender studies and trans studies. A prior relationship was established through the PAR program's initial workshops to build trust. Participants were informed of the researchers' goals and their positionality as allies in the IAP process. We used purposive and snowball sampling to ensure diverse representation within the trans community in Cali.

Data were analyzed using thematic analysis. An initial coding framework was developed and iteratively refined. Data saturation was reached when no new themes emerged from the focus groups. To ensure rigor, we employed triangulation with some participants to validate our interpretations.

### 2.7 Ethical considerations

This research was reviewed and approved by the Institutional Review Board (IRB) of the Faculty of Health Sciences of the Pontificia Universidad Javeriana Cali, Colombia, with approval certificate number 007–2021 dated June 26, 2021. Classified as research with minimal risk and in accordance with core principles of the Belmont Report; respect for persons, beneficence, and justice, and non-maleficence according to Colombian regulations (Resolution No. 008430 of 1993 of the Ministry of Health). This study was conducted in accordance with the ethical principles for medical research involving human subjects outlined in the Declaration of Helsinki (2013) and the International Ethical Guidelines for Health-related Research Involving Humans by the CIOMS (2016).

This research applied principles of reflexivity, positionality, and transdisciplinarity to understand the eating practices and beliefs of transgender women, following the framework of Participatory Action Research (PAR). The study emerged from needs that were community-validated and agreed upon by the community leader and co-investigator of the Research Program, as well as from situated recommendations at local, national, and global levels derived from scientific literature reviews and public policy guidelines.

All research activities were consensually designed and discussed within the community. Similarly, findings were shared through individual reports and both group and personalized discussion sessions, tailored to each participant's interests. The research process was co-created through spaces of dialogical participation, maintained under permanent critical reflexivity, and characterized by an explicit recognition of power/knowledge dynamics and situated privileges. This ensured the ethical construction of participatory and transdisciplinary knowledge.

It had a research protocol approved by the ethics committee and was developed in accordance with Colombian regulations. All participants received a printed informed consent form in Spanish, which was explained in person by two duly trained project researchers, who answered questions and allowed sufficient time for participants to decide whether to participate without any pressure. The written consent included specific authorization for audio recording of the discussion groups, the use of textual quotations using pseudonymization, and an express reminder that they could refuse to answer certain questions or withdraw from the study at any time without consequences. Prior to data collection for chain sampling, trans leaders were trained, and to minimize undue influence, the roles of community mediation and consent obtaining were separated. It was clarified that access to support or services did not depend on participation in the research. As minimal compensation for expenses, all participants were offered meals the day of data collection and a fixed transportation allowance (USD $2.80), without constituting a coercive incentive.

All data collection activities were carried out in closed and secure spaces at the Pontificia Universidad Javeriana Cali, ensuring privacy, confidentiality among peers, and the use of preferred names and pronouns. Confidentiality agreements were signed for the discussion groups, and a protocol for managing emotional distress was implemented, with the presence of a psychology professional who is an expert in gender and referral routes to psychosocial and nutritional support when necessary. Information management was carried out in compliance with Law 1581 of 2012 and Decree 1377 of 2013. Consent forms, field notes, and forms were physically filed in envelopes separate from the research data. Pseudonymized electronic databases and audio recordings were stored in a protected folder, under the custody of the principal investigator and subject to institutional IT security policies, with restricted access via password and confidentiality agreements for any secondary use for academic purposes.

We confirm that all participants in this study were aged 18 years or older. Age was verified through national ID cards during the initial screening and the informed consent process. The research instruments did not record names or any contact information that could link the responses to a specific individual. Databases were fully anonymized, with each participant assigned a unique alphanumeric code. All physical documents were securely stored in a locked filing cabinet within the Principal Investigator's (PI) office to ensure restricted access and maintain participant confidentiality.

## 3 Results

The results of the quantitative component will be described first, giving an account of the sociodemographic characteristics of the participating women and then their HEPI. The qualitative component will then be developed through the categories that emerged in the discussion groups.

### 3.1 Sample characteristics

According to Table 2, 81.8% of participants were aged 30 or over, on a low or middle socioeconomic level (48.5%; 51.5%), with a monthly income of less than 2 minimum monthly wages (87.9%). 91.0% had health insurance. 75.8% had basic education, and 72.7% lived unaccompanied. 87.9% reported working, and the most frequent jobs were hair stylist (36.4%).

**Table 2:** Sociodemographic characteristics of participants.

		Number of individuals	%
Age (Range: 23–66;43.24)	18–29	6	18.2
	30–45	11	33.3
	≥46	16	48.5
		33	100.0
Socioeconomic level	Middle	17	51.5
	Low	16	48.5
		33	100.0
Income level^a^	No salary	2	6.1
	Earns twice the monthly minimum wage or less	29	87.9
	Earns between twice or five times the monthly minimum wage	2	6.1
		33	100.0
Health insurance	Yes	30	91
	No	3	9
		33	100
Educational level	Technical/Technological	8	24.2
	Basic education (High school or lower)	25	75.8
		33	100.0
Living situation	Alone	9	27.3
	Accompanied	24	72.7
		33	100.0
Occupation	Call center	4	12.1
	Sex worker	2	6.1
	Web Cam model	4	12.1
	Stylist	12	36.4
	Other activities	7	21
	Unemployed	4	12.1
		33	100
Health condition diagnosed	Yes	17	51.5
	No	16	48.5
		33	100
Health condition diagnosed (*n* = 17)^b^	HIV	7	41.2
	Arterial hypertension (AH)	2	11.8
		16	100
Currently, or used to use HT	Yes	28	84.8
	No	5	15.2
		33	100
Who prescribed the HT (*n* = 28)	Self-medicating	20	71,4
	Health professional	8	28.6
	Total	28	100
Special diet or meal plan	Yes	9	27.3
	No	24	72.7
		33	100
Type of diet or special meal plan (*n* = 9)	Popular knowledge diet	2	22.2
	Increased intake of fruits and vegetables	2	22.2
	Reduction of sugars and fried foods	5	55.6
		9	100
Diet recommended by (*n* = 9)	Health professional	1	11.1
	Own initiative	8	88,8
		9	100
Receives or received treatment with supplements and/or nutritional supplements	Yes	17	51.5
	No	16	48.5
		33	100
Prescribed nutritional supplement (*n* = 17)	Self-medicating	13	76.4
	Health professional	4	23.5
		17	100

a A monthly minimum wage is approximately US $390.

b One reported being diagnosed and did not report her health condition.

Regarding health and nutrition, 51.5% reported having some form of illness, the most frequent being HIV infection (41.2%). Of the 84.8% that reported using or having used hormonal therapy [HT], 71.4% of them self-medicated. 27.3% followed a special diet during their gender transition, with an emphasis on reducing the consumption of sugars and fried foods (55.6%). In 88.8% of cases, changes in eating practices were made voluntarily. In turn, 51.5% of the population consumed nutritional supplements (Table 2).

According to the classification obtained with HEPI, 18 women (55%) were in the group with low levels of healthy eating practices; i.e., these women have a less diversified diet across the most recommended food groups and consume fewer recommended food groups more frequently. The highest proportion of women with unhealthy practices were in the middle social classes (12/17 70.5%), aged between 18 and 29 years (6/6100%), with low levels of education /14/25 56%), an income of less than two minimum monthly wages (16/29 55%), and those living in a couple (2/3 67%).

### 3.2 Bayes predictive probabilities

The conditional Bayes predictive probabilities were calculated to account for the different conditions established as factors that can modify women's eating practices. These probabilities can serve as a risk indicator to reveal unhealthy eating practices, as in Hoyos-Hernández et al. [25].

According to Table 3, belonging to the middle socioeconomic level, being under 29 years of age, having a low level of education, having an income of less than two minimum monthly wages, living with a partner, and always having a saltshaker on the dining table are factors that individually increase the probability of having unhealthy eating practices. Although all predictive probabilities are above 50%, the highest indicator of unhealthy eating practices is significant among people under 30.

**Table 3:** Estimated predicted probabilities of having unhealthy eating practices based on sociodemographic characteristics.

Feature	Probability of having a type of practice and RC^a^ 95_%_	
	Healthy	Unhealthy
Socioeconomic level		
Middle	31.58 (13.3–54.5)	68.42 (47.2–87.7)
Low	61.11 (39.3–81.0)	38.89 (18.3–62.2)
Age (years)		
18–29	12.50 (0.3–40.3)	87.50 (61.1–99.7)
30–45	61.54 (34.0–84.9)	38.46 (16.3–65.6)
≥46	50.00 (28.0–74.0)	50.00 (28.2–72.4)
Education		
High school or lower	44.44 (27.0–62.7)	55.56 (36.5–72.9)
Technical/Technological	50.00 (21.8–78.7)	50.00 (20.5–77.6)
Income		
Low–High	50.00 (13.0–82.6)	50.00 (14.0–85.2)
Middle	45.16 (26.6–63.1)	54.84 (37.8–70.9)
Living situation		
Alone	45.45 (18.6–73.5)	54.54 (24.0–82.1)
With partner	40.00 (6.9–80.9)	60.00 (19.4–93.9)
Relatives or others	47.83 (28.8–68.4)	52.17 (31.0–71.6)
Saltshaker on the table		
Yes	33.33 (9.6–62.3)	66.67 (34.1–91.2)
No	50.00 (31.6–67.9)	50.00 (31.2–66.9)

a Region of credibility.

### 3.3 Qualitative component

Twenty-two trans women participated in the discussion groups, divided into four groups of four to seven women each. Each meeting lasted approximately 120 min. The initial categories encompassing sociodemographic data, culinary and dietary practices, and food beliefs and practices related to gender transition were defined in the semi-structured protocol. Consistent with the principles of phenomenological design, two thematic categories were renamed and refined based on the participants' narratives: (1) *Food beliefs beneficial to health and the feminization process*, and (2) *Factors associated with food*. They focused on the discussion of foods and practices that they considered for and against the feminization of their gender expressions, as well as on the characteristics or circumstances of the environment that they perceive as influencing food consumption. In response to the first category, they expressed their ideas and convictions about food that affect their gender transition. The factors associated with food referred to the characteristics or circumstances of the environment that they perceive as influencing food consumption. The following is a description of the accounts of the women participants included in the previously indicated categories.

#### 3.3.1 Health-promoting dietary beliefs and the feminizing process

In this category, women said they consider foods that support their feminizing process and should be consumed during the hormonal process. In this regard, they say that smoothies with grapes, peanuts, spinach, turmeric, and guava strengthen the body's immune system and make them feel much better. In addition, they say that regularly eating fruit, vegetables, and protein-rich foods (such as chicken, eggs, and meat) can be beneficial, probably because the hormones in those animals support their health and the feminizing process.

*I drink green smoothies, I also do it for health, to detox, because I used to drink a lot of liquor. Or I drink herbs, or when I go shopping, I eat a lot of carrots and onions. (Transcription FG N° 3 - August 23, 2021).*

*Chicken has a lot of hormones, and that is why children can't eat a lot of it because it has a lot of hormones… well, when I take my hormones, I always have a glass of warm milk. (Transcription FG N°4 - August 23, 2021).*

In the discussion groups, their own beliefs also emerged, and those of their peers in the trans community, who think that drinking warm, boiled milk mixed with rum is a way to enhance the effects of hormones and strengthen the feminization of their bodies with much faster results.

*… She said that hormones exploded, that they worked better, liquor too, but those were myths, weren't they? (Transcription FG N° 1 - July 21, 2021).*

*The rum explodes the hormones. It's really good…you see the change faster. (Transcription FG N°4 - August 23, 2021).*

They also discussed several foods that should be avoided as they are not considered beneficial for their health. Regarding this, they mentioned the need to avoid fried foods due to trans fats, ultra-processed foods such as canned items or processed meats, fast food, and sweets. Furthermore, they relate healthy food or a healthy diet with preparations they make themselves, even if some of them do not enjoy cooking. For them, selecting and preparing their own food ensures better dietary practices. Some of the women report that they are careful about consuming certain foods and restrict their intake in the evenings to avoid weight gain and encourage weight loss.

*I like making salads and do not eat a lot of fried food. I eat what I feel like now, and then I eat it. I rarely eat processed meats; I don't eat them because I don't like them. I drink lactose-free milk… I also eat meat only once or twice a week; I prefer chicken, fish, things like that, but I do enjoy cooking. (Transcription FG N° 3–23 de Agosto 2021).*

*I cook because I must, and why is that? Because it is truly very difficult to go out and get healthy food, I have this thing where I like to take care of myself a little bit. That means I don't go out much to eat things like burgers or pizzas. Maybe I'll do it, but very sporadically, not all the time. So, the problem is that I come home and I have to prepare my own food because I am forced to, not because I want to. (Transcription FG N° 2–3 de Agosto 2021).*

*My fast is I have a good breakfast, and throughout the day I drink water, and then at a set time, 4 or 5 in the afternoon, I do eat something. But I do the fast for the one above (God), and for my health. (Transcription FG N° 2–3 de Agosto 2021).*

*Yes, I keep a list… I like to use gourmet oil or olive oil to avoid the cholesterol thing, to avoid trans fats. (Transcription FG N° 2–3 de Agosto 2021).*

Lastly, in this category, a discussion group recommended avoiding foods (in the Valle del Cauca culture) associated with cultural practices in the Colombian Pacific known as “aphrodisiacs”. They mentioned that these may increase testosterone levels, and in the case of trans women who are in hormone therapy, can mean a setback in their transition. The same holds for passion fruit, which has traditionally been associated with masculinity and virility.

*Well, I have heard about Borojó juice. It is supposed to be an aphrodisiac. I have had it, but I don't… I don't know how real it is, it could be real, you know what I mean? But I don't know… Because perhaps it causes the growth of male hormones. (Transcription FG N° 2 - August 3, 2021).*

*Well, you hear about passion fruit, that you shouldn't eat passion fruit because it encourages the growth of testosterone. (Transcription FG N° 2 - August 3, 2021).*

#### 3.3.2 Factors associated with food consumption

Regarding the factors associated with food consumption, the women detailed their personal narratives, addressing issues related to socioeconomic circumstances, their food consumption schedules, work activity, the use of psychoactive substances, and hormone therapy for their feminization process.

Those who mentioned work-related aspects associated it with being sex workers and the need to adopt dietary practices that ensure energy and hygiene. Additionally, some aspects of the work environment that the women mentioned relate to their access to certain foods, typically fried or fast food. This often leads to an increased intake of these items or a lack of options for choosing healthier foods.

*If a girl works, for example, she can be a webcam model or a sex worker, then she has to work a lot of shifts… It's tiring because they have to work several times, so I imagine that they burn a lot of energy and that makes them eat more. So that's why we say they eat like men because men use more energy. (Transcription FG N° 1 - July 21, 2021).*

*The thing is, since I am a webcam model, one has to use dildos, so for reasons of ethics and hygiene, I prefer not to eat. That is, it's like a preparation for work; just as one prepares with makeup and the clothes they wear, their body also has to prepare for that type of activity. So, it's already like a ritual—I never eat anything before going to work. (Transcription FG N° 1 - July 21, 2021).*

*Where I work there are a lot of fast food options: pizzas, hamburgers, carne a la llanera (Llanos-style grilled beef), lechona (stuffed pork), and desserts. (Transcription FG N° 2 - August 3, 2021).*

On the other hand, regarding economic aspects, some of the women reported difficulty covering the cost of certain foods they consider healthy, such as high-quality cooking oil, seafood, and quinoa.

*Well, personally, seafood is expensive, and the preparation is also expensive, so one doesn't consume it. What is accessible is the fish, which already comes filleted for you to buy, but things like cazuela (seafood stew) and other things like that, no. (Transcription FG N° 2 - August 3, 2021).*

*Sometimes one has the money to buy food, but they often restrict themselves, right? Because they have to pay this, they have to pay that. “Oh, I wish I could eat a quarter of a chicken, but no, tomorrow I have to buy such-and-such...” (Transcription FG N° 2 - August 3, 2021).*

Finally, other factors related to eating practices include the consumption of psychoactive substances and hormone therapy. For instance, the use of certain psychoactive substances, such as marijuana and cocaine (perico), was reported to increase their appetite and food intake. This also occurs during the first few days or even the first month after initiating hormone therapy. In this regard, it is essential to note that some of the participating women access pharmaceuticals related to feminine gender expression, such as estrogens, contraceptive pills, or injections, through their own means and without specialized medical supervision. These instances often involve irregular, sporadic consumption, or consumption only when they feel it is time to strengthen their feminine gender expression process.

*Something to bear in mind is that girls who consume substances have a greater appetite. But that depends. When they consume chemicals such as perico, the drink that takes away your appetite, then the next day they wake up with a hangover, and the only thing they want is liquid, which is not good for their bodies. (Transcription FG N°4 - August 23, 2021).*

*That is, when one takes hormones, one injects oneself, and it makes one hungrier. (Transcription FG N° 1 - July 21, 2021).*

*Yes, I believe the hormone itself lasts for a month, right? So, during that month, you do get hungrier. When I usually take hormones, yes, and one even gains more weight and everything. (Transcription FG N° 1 - July 21, 2021).*

## 4 Discussion

Based on the objective of this research, which sought to describe and categorize the eating practices and beliefs of trans women, we have highlighted that the eating practices of the participants have a predictive probability that is not very good for their health. This applies to those with the following characteristics: low socioeconomic level, under 29 years of age, low level of education, income of less than two minimum monthly wages (USD$390), living as a couple, and using a saltshaker more often at the dining table. These factors account for the social determinants that have a direct impact on the behavior, health, and illness processes of trans women.

Colombia, which, according to Law 1622 of 2013, includes individuals aged 14 to 28 [26]. This stage of life is characterized by the pursuit of autonomy, access to education and employment, and the consolidation of social relationships ^20^. In this study, young participants reported difficulties accessing educational and employment opportunities and experiencing housing instability. These findings align with data from the Colombian National Statistics Department [27], which indicates that young people (under 30 years of age) face similar difficulties. Nevertheless, it is emphasized that, due to limited data availability, it wasn't possible to compare the results of the trans women population in this study with national data on the trans population. Despite this, literature does state that particularly those with diverse gender identities face high levels of social exclusion, unemployment, and limited access to basic services. This convergence highlights the importance of interpreting dietary practices among trans women through a social determinants of health perspective.

Added to this, in Colombia, there is a high rate of casual labor among young people, low labor-market participation, higher unemployment, and lower employment rates 20, and, at a psychological level, a lower perception of risk [28,29]. These factors negatively impact their income, health, well-being, and self-care, which, in this case, is related to their eating practices.

However, middle-aged women (40–59 years) have a balanced well-being and may be more prone to developing illnesses. This age group plays a fundamental role in the lives of younger and older people and is considered a bridge and caregiver population between generations [30]. From the quantitative and qualitative results, it is clear that women in this age group are crucial in transmitting knowledge about their trans life experiences. They influence younger people on subjects such as hormone therapy and nutrition. Other studies have reported trans peers as a significant support network for well-being, health, and illness processes [8,[31], [32], [33]].

As a consequence of the barriers to health care, it is common for trans women to receive little information from health and nutrition professionals. This leads them to base their eating practices on the information they have gathered through popular knowledge and peer processes. Thus, this study identifies the importance of women in building knowledge within their own community from their trans life experiences. This behavior is the result of the barriers trans women face when trying to access trained professionals to respond to their needs. Evidence of this is the high prevalence of self-medicated hormone therapy (84.8%) and its potential cardiovascular risks [9,10,12,34].

According to the classification obtained using the HEPI, 18 (55%) of the participants have unhealthy eating practices, i.e., they have a less diversified diet across the most recommended food groups and consume the least recommended food groups more often. It is important to note that 51% of participants use nutritional supplements, compared to 8.1% of the Colombian population, according to the ENSIN 2015 [20]. This fact reinforces the validation of practices among themselves to address their needs. Another aspect to consider is the possible risks of adverse effects that may be caused by self-medication. In addition, 58% of trans women eat outside their home, compared to 40.4% of the adult population in Colombia, according to the results of the ENSIN [20]. Participants' fast-food consumption is the same as that of the Colombian adult population at 54.5%. Still, the frequency of consumption is much higher among trans women, 61% of whom eat fast food daily, compared to three times a month for the adult Colombian population.

The narratives of trans women in Cali reveal that their eating practices and beliefs are the result of a complex and multidimensional process that integrates structural and systemic aspects related to gender, socioeconomic context, and health processes [8]. In this regard, they hold a strong belief that links eating practices to gender expression and decisions about which foods to avoid, such as fried foods, ultra-processed products, sugars, and those considered aphrodisiacs. They also report that the use of nutritional supplements, fasting, and alcohol consumption combined with hormone therapy promotes their health, hygiene, and the feminization process.

Although these beliefs may have positive effects on their health, others could be harmful, such as certain alcoholic drinks. At a psychological level, some of these factors may affect their mental health and well-being. This may be due to their concern and constant search for congruence between their gender identity and gender expression.

Studies report that, for those who desire it, the physical transition related to gender expression—for example, through hormone therapy or gender-affirming surgeries—positively contributes to individuals' well-being. These procedures are associated with lower prevalences of depression, anger, social anxiety, and generalized anxiety, as well as increased feelings of happiness, satisfaction with one's own body, a greater sense of connection with oneself and others, and feelings of authenticity [8,32]. This relates to the fact that gender expression aligns with one's gender identity, and those eating practices are fundamental to health and body image processes.

Eating beliefs and practices are related to hegemonic constructions of femininity and health. These gender constructions permeate all processes of education, primary and secondary socialization, media, and social networks, generating intense psychological comparisons and tensions among women, which increases body dissatisfaction [8,35] In this regard, the trans category expands these hegemonic notions and gender binaries through the affirmation of their identities, their desires in concrete social experiences and practices [36]. In this way, these women are in constant tension between social reproduction (through socialization) and the management of agency (through resistance and recognition), contributing in some dimensions to social destabilization and transformation.

However, people should enjoy a healthy diet at different stages of life, including foods that meet their energy and nutritional needs based on age, gender, physiological state, and any health conditions. Their diet should be complete, balanced, sufficient, adequate, and safe [14]. In light of the above, it can be seen that the trans women participants require continuous support to deepen their beliefs and receive advice on healthy eating, to reinforce the positive and protective effects on their health. This is especially the case with their hormone therapy (as part of their feminizing process), which may cause cardiovascular disease [[10], [11], [12]].

In this regard, it is important to take action to improve these behaviors (given the inequalities and concerning indicators among the trans population) and to join efforts aligned with the Sustainable Development Goals, the FBDG, and recommendations for the prevention and control of chronic noncommunicable diseases. It has been highlighted that the trans community represents the most critical morbidity in the world [37,38]. To support the needs of this population, joined-up and interdisciplinary work is required between the health sciences and health psychology (the latter falling within the social and human sciences) to propose projects and interventions with a differential and rights-based approach.

## 5 Limitations and future research

The limitations of this study are presented at multiple levels. First, the scarcity of scientific literature focused on the diet and nutrition of transgender women limited the possibilities for comparison and triangulation with direct conceptual and empirical references. Second, in the quantitative component, the construction of the HEPI and the use of the Bayesian paradigm allowed for an analytical and probabilistic approach to dietary patterns; however, due to the small sample size and the use of chain sampling, the results should be interpreted from an exploratory and contextual perspective, without generalizing to the population.

In the qualitative component, although the findings provide descriptive density and conceptual depth, they reflect the experiences of the participating women and, to a greater extent, of people with similar sociodemographic characteristics. In this sense, the transferability of the results is not based on statistical extrapolation, but rather on analytical transferability, as they document dietary practices and health beliefs constructed in contexts of structural inequality, discrimination, and limited access to health services. These dynamics may be relevant for understanding similar situations in other trans populations and historically underserved groups that share similar conditions.

Finally, the main challenges for future research on food and nutrition in the trans population include the development of longitudinal and multicenter studies that allow for an integrated analysis of dietary practices, gender-affirming hormone therapy, and its influence on metabolism, body composition, and cardiometabolic risk throughout the life course. Gaps remain in the characterization of diet quality, food insecurity, and risky eating behaviors, especially in Latin American contexts marked by structural inequalities and barriers to access to trans-affirmative health services. In this context, there is a need to develop gender-sensitive tools and evaluate culturally adapted nutritional interventions aimed at generating contextualized and transferable evidence that contributes to the development of inclusive public policies.

## 6 Conclusion

The results of this study reflect and provide evidence of the social, health, food, and nutritional needs of trans women in Colombia. Trans identities face several structural barriers in Colombia that perpetuate inequalities and accentuate the results of negative health and well-being. Studies such as this make these situations visible and reiterate the need for commitment to developing comprehensive, intersectoral, and intersectional actions that reduce these gaps in differential ways. Actions to provide protection and guarantees for the health and the welfare of the trans community are urgently needed.

The findings highlight the need to incorporate Primary Health Care (PHC) approaches with a gender perspective, ensuring timely access and trans-affirmative care. Within this framework, nutrition and dietetics professionals play a fundamental role in understanding nutrition and food consumption through the lens of the social determinants of health and with a gender focus. Furthermore, it is essential to continue transforming hegemonic social beliefs and representations regarding gender and femininity to create truly inclusive health environments and a society that embraces the very diversity that shapes, nourishes, and represents it.

The academic and scientific community should also demand the construction of knowledge that includes the voices of people, interactions based on trust, and the non-pathologization and medicalization of people with trans life experiences. Such action is needed to continue building crucial scientific knowledge horizontally with the trans population itself, especially given the limited studies on nutrition and food for this population.

The HEPI index, designed for this research, can be used to identify, descriptively, the characteristics associated with people's eating practices across populations. Its design included a heuristic construction process based on information from specialists, because the study's small sample size did not allow the use of more elaborate methods such as multivariate statistical analysis.

Finally, one of the innovations and strengths of this research was the use of a mixed design. This made it possible to include empirical-analytic, historical, and interpretive approaches, which can enrich existing studies of the trans community and their food practices and beliefs, which are scarce. The discussion groups conducted in this research enabled broader information and understanding of this topic and provided a more robust, focused baseline of trans life experiences to consolidate transdisciplinary evidence.

## Contribution to the sustainable development goals

This study aligns with the Sustainable Development Goals (SDGs) by providing empirical evidence on dietary practices and health-related conditions in transgender women. It contributes to SDG 3 (Good Health and Well-being) by generating knowledge relevant to understanding nutritional factors that may influence health outcomes. It relates to SDG 5 (Gender Equality) by incorporating an analytical approach that recognizes gender diversity in the study of dietary practices. It is also linked to SDG 10 (Reduced Inequalities) by analyzing differences in practices and social conditions associated with food. Although non-probabilistic sampling was used, the findings contribute to guiding more inclusive and rights-based institutional policies and practices, in line with the principles of SDG 16.

## CRediT authorship contribution statement

**Ana Lucia Valenzuela-Gallego:** Writing – review & editing, Writing – original draft, Visualization, Validation, Supervision, Project administration, Methodology, Investigation, Formal analysis, Conceptualization. **Paula Andrea Hoyos-Hernández:** Writing – review & editing, Writing – original draft, Visualization, Validation, Supervision, Methodology, Investigation, Formal analysis, Data curation, Conceptualization. **Laura Lucia Dominguez-Barrios:** Writing – review & editing, Writing – original draft, Visualization, Software, Methodology, Formal analysis, Data curation. **José Rafael Tovar-Cuevas:** Writing – review & editing, Writing – original draft, Visualization, Software, Methodology, Formal analysis, Data curation.

## Funding

This article is resulted from the research project “Body composition, self-perception of body image, eating practices, and beliefs in transgender women living in Cali,” approved in the 2019 Internal Funding for Research Projects call by the Pontificia Universidad Javeriana, Cali, Colombia with institutional funding Investigar PUJ1528.

The funder had no role in the design of the study, the collection, analysis, interpretation of data, nor in the writing of the manuscript.

## Declaration of competing interest

The authors declare no possible conflicts of interest.

## Data Availability

The research protocol was not prospectively registered in open access repositories, as the study was conducted in accordance with an institutionally approved protocol and prospective public registration was not contemplated during the design phase. Due to the sensitive nature of the information collected and the ethical commitment to protect the privacy, confidentiality, and safety of the trans women participants, quantitative data and qualitative transcripts are not publicly available. Data availability was not contemplated in the approved protocol, and access to anonymized information may only be considered on an exceptional basis, subject to ethical approval and upon reasonable request to the corresponding author.

## Appendix A Supplementary data

Supplementary material 1 STROBE checklist completed by the authors, detailing compliance with the recommended items for reporting observational studies. Supplementary material 1

Supplementary material 2 COREQ checklist completed by the authors, documenting compliance with the recommended criteria for reporting qualitative research. Supplementary material 2
