# Supplementary material for: Inequalities, eating practices and beliefs among transgender women in Colombia: Mixed approaches in research
Source: Dialogues Health. 2026 Feb 26;8:100287. doi: 10.1016/j.dialog.2026.100287 (PMC12972529; doi:10.1016/j.dialog.2026.100287)
Supplement: Supplementary material 1 — STROBE checklist completed by the authors, detailing compliance with the recommended items for reporting observational studies. [file mmc1.pdf]

Inequalities, eating practices and beliefs among transgender women in Colombia: mixed approaches in research

STROBE Checklist

Cross-sectional Quantitative Component

STROBE Item	Reporting requirement	Section in manuscript
1a–1b	Study design and abstract	title in the manuscript and abstract
2–3	Background and objectives	Introduction
4	Study design (cross-sectional, observational)	Methods - Design
5	Setting, location, and data collection period	Methods - Procedure
6a	Eligibility criteria	Methods - Participants
7–8	Variables, instruments, and data sources	Methods
9	Potential sources of bias	Discusión and limitations and future research sections
10	Rationale for sample size	Methods - Participants - Data analysis
11	Handling of quantitative variables	Methods - Data analysis
12a	Statistical methods	Methods - Data analysis
12c	Handling of missing data	Methods - Data analysis
12d	Sensitivity analyses	NA
13–14	Participants and descriptive data	Results
15–16	Outcome measures and estimates	Results
17	Key results	Discusión
18	Study limitations	Limitations and future research
19	Interpretation of results	Discusión
20	Generalisability / transferability	Limitations and future research
22	Ethical approval and informed consent	Ethical considerations

Ana Lucia Valenzuela Gallego
