# Supplementary material for: Inequalities, eating practices and beliefs among transgender women in Colombia: Mixed approaches in research
Source: Dialogues Health. 2026 Feb 26;8:100287. doi: 10.1016/j.dialog.2026.100287 (PMC12972529; doi:10.1016/j.dialog.2026.100287)
Supplement: Supplementary material 2 — COREQ checklist completed by the authors, documenting compliance with the recommended criteria for reporting qualitative research. [file mmc2.pdf]

Inequalities, eating practices and beliefs among transgender women in Colombia: mixed approaches in research

COREQ checklist

qualitative method

Item	COREQ domain	Description	Reported in manuscript
**Domain 1 Research team and reflexivity**			
1	Personal characteristics	Interviewer/facilitator	Methods – Qualitative component
2	Personal characteristics	Credentials	Methods – Qualitative component
3	Personal characteristics	Occupation	Methods – Qualitative component
4	Personal characteristics	Gender	Not explicitly reported
5	Personal characteristics	Experience and training	Methods – Qualitative component
6	Relationship with participants	Relationship established	Methods – Procedure / Ethical considerations
7	Relationship with participants	Participant knowledge of interviewer	Methods – Qualitative component
8	Relationship with participants	Interviewer characteristics / positionality	Ethical considerations
**Domain 2: Study design**			
9	Theoretical framework	Methodological orientation	Methods – design
10	Participant selection	Sampling method	Methods – Procedure
11	Participant selection	Method of approach	Methods – Procedure
12	Participant selection	Sample size	Methods – Procedure
13	Participant selection	Non-participation	Not reported
14	Setting	Setting of data collection	Methods – Procedure
15	Setting	Presence of non-participants	Not reported

16	Data collection	Description of sample	Results – Participant characteristics
17	Data collection	Interview guide	Methods – Instruments
18	Data collection	Repeat interviews	Not applicable
19	Data collection	Audio/visual recording	Methods – Ethical considerations
20	Data collection	Field notes	Not reported
21	Data collection	Duration	Not explicitly reported
22	Data collection	Data saturation	Methods – Instruments / Qualitative analysis
**Domain 3: Analysis and findings**			
23	Data analysis	Number of data coders	Methods – Qualitative analysis
24	Data analysis	Description of coding tree	Not reported
25	Data analysis	Derivation of themes	Methods – Qualitative analysis
26	Data analysis	Software	Methods – Qualitative analysis (Atlas.ti 22)
27	Data analysis	Participant checking	Ethical considerations
28	Reporting	Quotations presented	Results
29	Reporting	Data and findings consistent	Results / Discussion
30	Reporting	Clarity of major themes	Results
31	Reporting	Clarity of minor themes	Results
32	Reporting	Participant feedback on findings	Ethical considerations

Ana Lucia Valenzuela Gallego
